# A suspected case of hepatic reactive lymphoid hyperplasia in which EUS–fine needle aspiration contributed to the diagnosis

**DOI:** 10.1097/eus.0000000000000092

**Published:** 2024-12-30

**Authors:** Yuya Sato, Tsuyoshi Suda, Yasunori Sato, Kiichiro Kaji, Shuichi Terasaki

**Affiliations:** 1 Department of Gastroenterology, Kanazawa Red Cross Hospital, Kanazawa, Japan; 2 Department of Human Pathology, Kanazawa University Graduate School of Medicine, Kanazawa, Japan.

**Figure 1:**
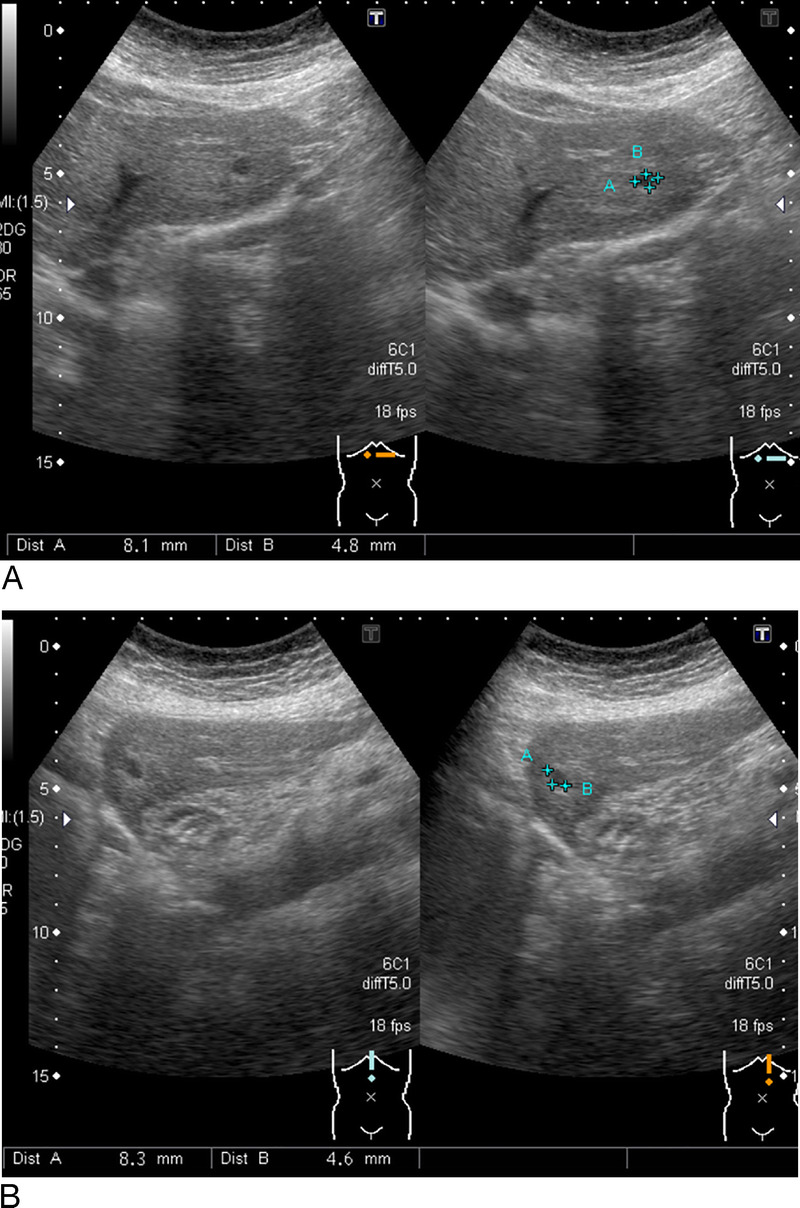
Ultrasound shows an 8-mm hypoechoic mass in the left lateral section of the liver on initial evaluation (A, B).

**Figure 2:**
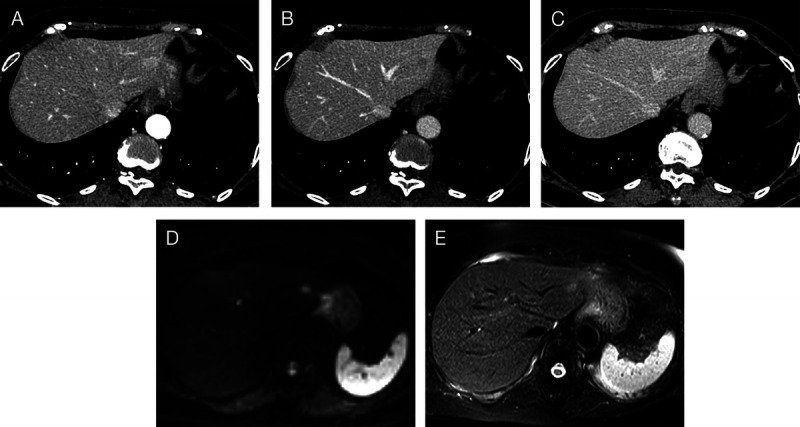
Contrast-enhanced computed tomography shows homogeneous enhancement in the arterial dominant phase (A) and wash-out with suspicion of slight perinodular enhancement in the portal (B) and late phases (C). Magnetic resonance imaging shows high-signal intensity on diffusion-weighted imaging (D) and slight signal intensity on T2-weighted imaging (E).

A 70-year-old female with a 10-year history of an incidentally diagnosed 8-mm hypoechoic lesion in the left lateral section of the liver on abdominal ultrasonography [Figures 1A and B] was evaluated for its gradual enlargement. Contrast-enhanced computed tomography showed homogeneous enhancement in the arterial-dominant phase and wash-out with suspicion of slight perinodular enhancement in the portal and late phases [Figures 2A–C]. Magnetic resonance imaging revealed high-signal intensity on diffusion-weighted imaging [Figure 2D] and slight signal intensity on T2-weighted imaging [Figure 2E].

**Figure 3:**
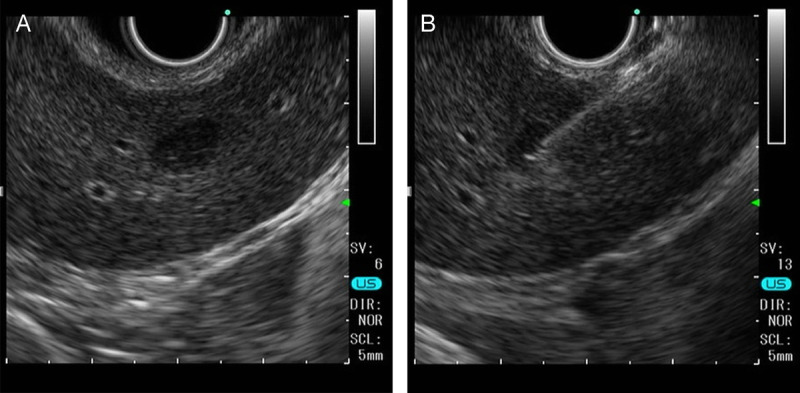
EUS shows a hypoechoic mass in the left lateral section of the liver (A). EUS–FNA was performed via the stomach (B).

Hepatocellular carcinoma (HCC) could not be ruled out based on the imaging results, and given the lesion's location, EUS–fine needle aspiration (EUS–FNA) was performed. A space-occupying lesion (SOL) was observed in the left lateral section of the liver [Figure 3A], and a 22-gauge needle was used to obtain a sample via the stomach [Figure 3B]. No complications occurred post-biopsy.

**Figure 4:**
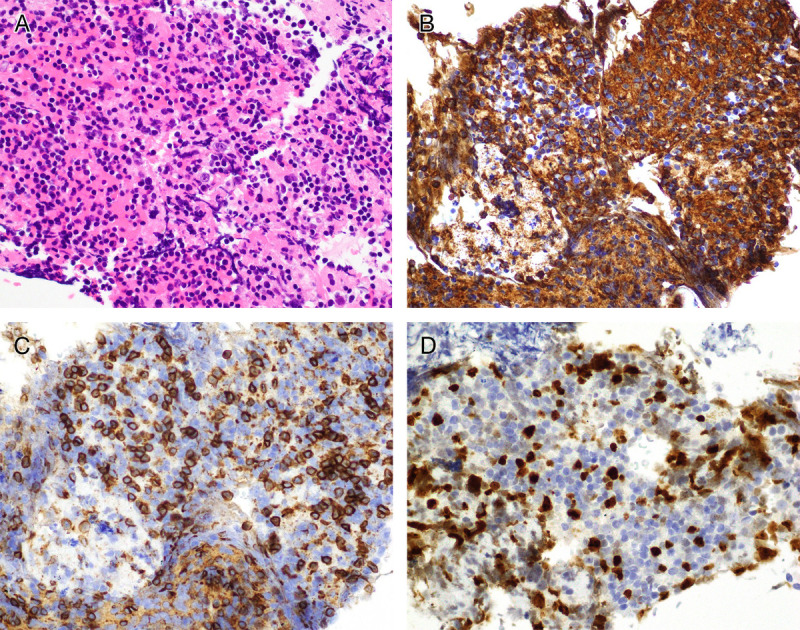
Hematoxylin and eosin staining (A), immunostaining of CD20 (B), CD3 (C), and Ki67 (D) of the sample are shown (magnifications, ×400).

Histopathological examination findings showed lymphocyte clusters without atypia [Figure 4A], consisting predominantly of CD20-positive B cells [Figure 4B] and some CD3-positive T cells [Figure 4C]. In situ hybridization studies for kappa and lambda chains showed no light chain restriction, and Ki67-labeling yielded positive results (38%; Figure 4D). No lymphoepithelial lesion of the bile ducts representing a histological feature of MALT lymphoma was observed in the specimen. Taken collectively, a histological diagnosis of hepatic reactive lymphoid hyperplasia (RLH) was strongly suspected. This is consistent with studies on hepatic RLH reporting relatively high Ki-67 indices, with a mean value of 40%, indicative of the lesion's reactive proliferative nature.^[1–4]^

Following EUS–FNA and pathological diagnosis, the patient underwent observation and monitoring. Despite over 1.5 years of imaging follow-up, the lesion showed no significant change. Imaging diagnosis of hepatic RLH can be challenging, with approximately 60% of cases being misdiagnosed as HCC or cholangiocarcinoma.^[5]^ In such cases where imaging findings are suggestive of malignancy, surgical resection may be necessary for definitive diagnosis.

This case highlights the utility and safety of EUS–FNA for obtaining a histological diagnosis of hepatic SOLs, especially for lesions localized in the left liver lobe.
